# Surgical aspects related to hereditary pancreatic cancer

**DOI:** 10.1007/s10689-024-00384-1

**Published:** 2024-04-25

**Authors:** Elisabeth Maurer, Detlef K. Bartsch

**Affiliations:** Department of Visceral-, Thoracic- and Vascular Surgery, Philipps University Marburg, 35043 Baldingerstrasse, Marburg, Germany

**Keywords:** Hereditary pancreatic cancer, Surveillance, Indication for surgery, Extent of surgery

## Abstract

The goal of surveillance programs for individuals at risk (IAR) from familial pancreatic cancer (FPC) families or families with other inherited tumor syndromes predisposing to the development of pancreatic adenocarcinoma (PDAC), such as hereditary pancreatitis or Peutz-Jeghers syndrome, is the dectection and consecutive curative resection of early PDAC or even better its high-grade precursor lesions. Although the indication for surgery is quite established, the extent of surgery is not well defined due to the lack of evidence-based data. In addition, multiple factors have to be taken into account to determine an optimal personalized surgical strategy. This holds especially true since pancreatic surgery is associated with a relatively high morbidity and might impair the quality of life significantly. In this article the surgical aspects in the setting of hereditary PDAC are discussed.

## Introduction

According to the Cancer of the Pancreas Screening (CAPS) consensus the primary goal of pancreatic surveillance is to prevent death from pancreatic cancer (PDAC) and ideally by preventing its development by identifying and treating its high-grade precursor lesions [1, 2]. Many factors should be considered when deciding about pancreatic surgery in individuals at risk (IAR), especially since the evidence in this setting is limited and recommendations from experts regarding the surgical approach are often only based on limited evidence. In case of screening detected suspicious pancreatic lesion(s), the IARs individual estimated risk for PDAC based on gene variant status and family history, age, comorbidities, life expectancy, compliance as well as the risk of any surgical procedure should be considered. Decision-making is best undertaken by an experienced, multidisciplinary expert team [1]. When surgery is indicated, it is best performed at a high-volume center, since several studies have shown that the case volume directly correlates with the surgical outcomes [2, 3].

### Indication for surgery

#### Proven PDAC or suspicious solid lesion(s)

In general, IAR should undergo pancreatic resection for almost the same indications as individuals without a known familial/genetic risk according to established guidelines [1, 4–6]. A screening-detected, resectable PDAC without distant metastases should be resected. A borderline resectable PDAC should nowaday undergo neoadjuvant treatment (e.g. chemotherapy with Folfirinox) and secondary resection, if feasible. Unambiguous solid lesions whose size is ≥ 0.5 cm or which can be represented in multiple imaging modalities are suspicious of malignancy and should be removed, if additional evaluation (e.g. EUS-guided biopsy) does not yield a definitive preoperative diagnosis [1, 2].

#### Cystic lesions

Cystic lesions, especially so called “imaging” branch-duct type (BD)-IPMN, are detected in more than 50% of IAR, but only a minority of these cysts become malignant during up to 5 years follow-up in reported screening programs [7–9]. The predictive value of imaging to detect neoplastic changes in a pancreatic cyst is limited. Hence, there is a high chance for an unnecessary pancreatic resection, if the threshold for surgery is set too low [10, 11]. However, a recent European evidenced-based guideline defined with regard to the risk of malignant transformation absolute and relative indications for surgery in cystic lesions, which should also be applied to IAR (6, Table 1). Absolute indications for surgery in IPMN are positive cytology for malignancy or high-grade dysplasia, a solid mass component, jaundice, enhancing mural nodule ≥ 5 mm and main pancreatic duct (MPD) diameter ≥ 10 mm (Table 1).

**Table 1:** Absolute and relative indications for surgery in IPMN according to the European Study Group on Cystic Tumors of the Pancreas [6]

Absolute Indication	Relative Indication	FaPaCa modification in FPC
positive cytology for malignancy or high-grade dysplasia	Growth-rate > 5 mm/year	None
solid mass component	Increased levels of serum CA19-9*	None
jaundice	Cyst diameter ≥ 40 mm	Cyst diameter > 20 mm, > 5 cysts > 5 mm
enhancing mural nodule ≥ 5 mm	New onset diabetes	No indication for itself
MPD diameter ≥ 10 mm	Acute pancreatitis caused by IPMN	None
	Enhancing mural nodule (< 5 mm)	none

*- in the absence of jaundice, MPD- main pancreatic duct, IPMN – intraductal papillary mucinous neoplasia

**Fig. 1:**
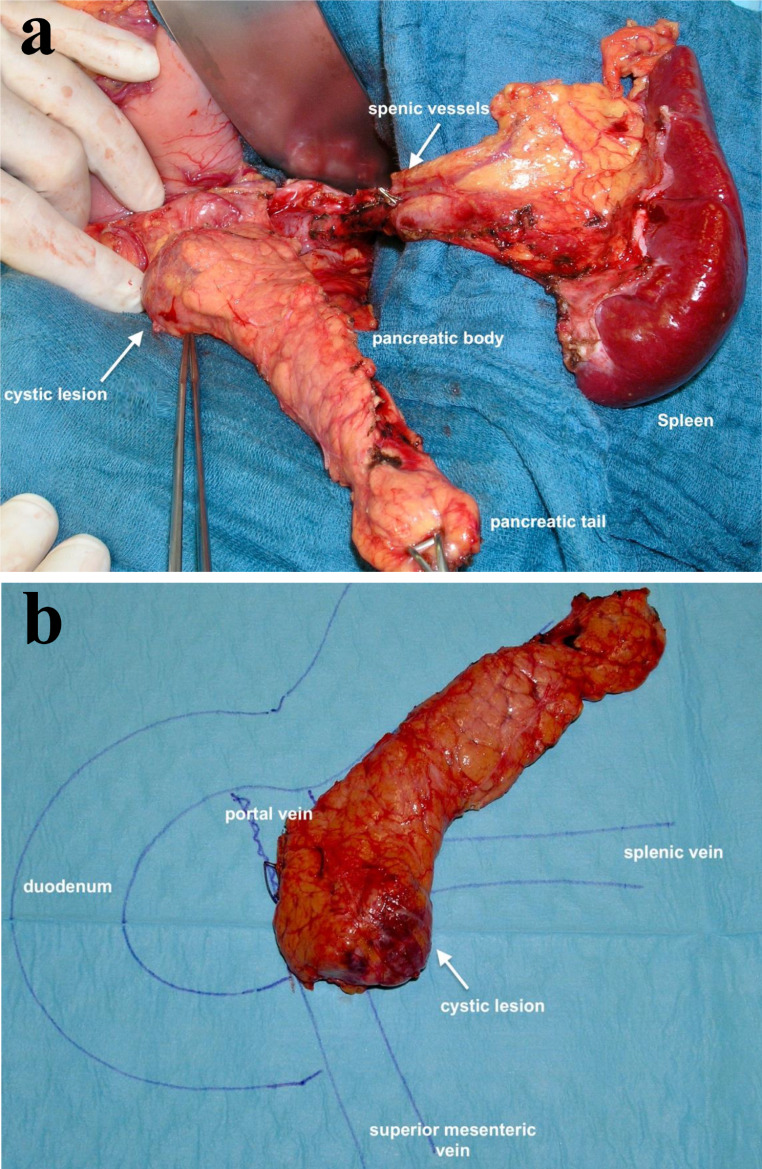
Vessel- and spleen-preserving distal pancreatic resection for a cystic lesion progressive in size, histopathology revealed a serous cystic neoplasm (SCN) (Department of Visceral-, Thoracic- and Vascular Surgery, Philipps-University Marburg). **A** Intraoperative situs. **B** Resected specimen

The relative indications should be adopted to the personal risk of the IAR, including affected members in the family, the presence of high-risk mutation, age and health status. The FaPaCa group (National Case Collection for Familial Pancreatic Cancer in Germany) indicates surgery also in IAR with pancreatic cyst > 20 mm or more than 5 cysts > 5 mm (Table 1) since these often indicate coexisting multifocal PanIN2/3 lesions [12] (Fig. 1).

Nowadays all types of pancreatic resections can be performed minimally invasive, especially with the robotic technique. In experienced centers the oncological results seem not to be inferior to the conventional technique, but the access trauma and general complications are reported to be lower [13–15].

### Prophylactic extension of resection

During the CAPS consensus conferences [1, 3] the management of IAR with resected lesions was controversely discussed, particularly how the intraoperative and final pathology results, including margin status, should influence operative treatment. Most experts agreed that in case of intraoperatively proven PDAC further resection up to total pancreatectomy should be performed to achieve a R0 resection of the tumor. The FaPaCa group is more aggressive. In case of intraoperatively proven PDAC they prefer extension of the resection to total pancreatectomy independent of the resection margin. They also will proceed with resection even up to total pancreatectomy until no high-grade PanIN/IPMN could be detected at the resection margin.

Intraoperatively, further pancreatectomy (including total pancreatectomy) should not be performed in IAR with only PanIN2 in the resected specimen, nor if PanIN2 or low-grade IPMN are present at the resection margin or in the resected specimen [1, 3]. The presence of PanIN3 should be dealt with in consideration of the overall medical condition and life expectancy of the patient [16]. The FaPaCa group will proceed to total pancreatectomy, if the intraoperative frozen section describes multifocal PanIN 3 lesions in the absence of PDAC. It is important to know, however, that it is sometimes very difficult to grade PanIN in intraoperative frozen Sections. [3]. Therefore, the presence of higher grade PanIN oder IPMN might first be stated in the definitive pathology report. Most experts agreed that redo operations for further resection of the pancreas to remove PanIN2 at the margin or because of uni- or multifocal PanIN2 lesions anywhere in the resection specimen should not be performed [1, 3]. Multiple scenarios for consideration of further pancreatectomy, including the management of PanIN3 lesions at the margin or anywhere in the resection specimen did yet not reach consensus [1, 3]. PanIN3 lesions at the resection margin in non-familial patients treated for PDAC did not affect postoperative course [16], but this remains yet unclear for PanIN3-lesion in the absence of PDAC. Most experts recommend follow-up imaging less than 6 months after surgery, if there was any PanIN3 lesion in the resected pancreas of IAR without PDAC [3]. The FaPaCa group, however, recommends completion pancreatectomy in this situation, since they have experienced that all 8 IAR with this condition remained free of pancreatic disease up to 131 months after surgery with an acceptable quality of life [17].

### Prophylactic pancreatectomy

The vast majority of experts agree that there is generally no indication for prophylactic pancreatectomy in asymptomatic IAR without any imaged lesion [1, 3, 18]. The complication rate of this procedure is about 30–40%, and the mortality around 1–6%, even when performed minimally invasive [19]. In addition, the consequences of exocrine and endocrine pancreatic insufficiency impair the quality of life sustainably [20]. Nevertheless, a Dutch group recently reported the PROPAN programme, which provides a conceptual and informative framework with decision tables for both IAR and physicians who wish to discuss prophylactic total pancreatectomy. This programme includes preoperative counselling, weighing the pros and cons between the reduction in PDAC risk and the risks and long-term consequences of total pancreatectomy, as well as the uncertainty regarding lifelong surveillance as an alternative management approach [21]. These considerations are important, since few IAR have serious cancer fear and therefore consider prophylactic pancreatectomy as it was the case in 2 IAR of the FaPaCa cohort [17].

An established exception to indicate a total pancreatectomy are symptomatic patients with hereditary pancreatitis (HP) and PRSS1 germline mutations since those have a PDAC lifetime risk of up to 40% [22, 23]. In symptomatic HP patients without suspicion lesions on imaging, however, endoscopic treatment is the most useful in patients with pancreatic duct lithiasis, obstruction, and dilation. It should be the first-line option, because it is less invasive than surgery. Surgery such as drainage operations (Puestow’s, Partington-Rochelle’s, Duval’s procedures), resectional operations (partial and subtotal or total pancreatectomies), resections with extended drainage (Beger’s, Frey’s procedures) should be the first-line option in patients for whom endoscopic treatment has failed or in those with a pancreatic mass with suspicion of malignancy. In general, total pancreatectomy should be considered in patients who have failed other operations or in patients with small duct or minimal change disease [24]. In these patients total pancreatectomy can be combined with autologous islet transplantation (see below).

### Total pancreatectomy with autologous islet transplantation (TPAIT)

Total pancreatectomy with autologous islet transplantation (TPAIT) was already decribed in 1980 by Najarian et al. for chronic pancreatitis [25] and became thereafter a rare, but established procedure in patients with advanced symptomatic chronic pancreatitis, including HP [26].

**Fig. 2:**
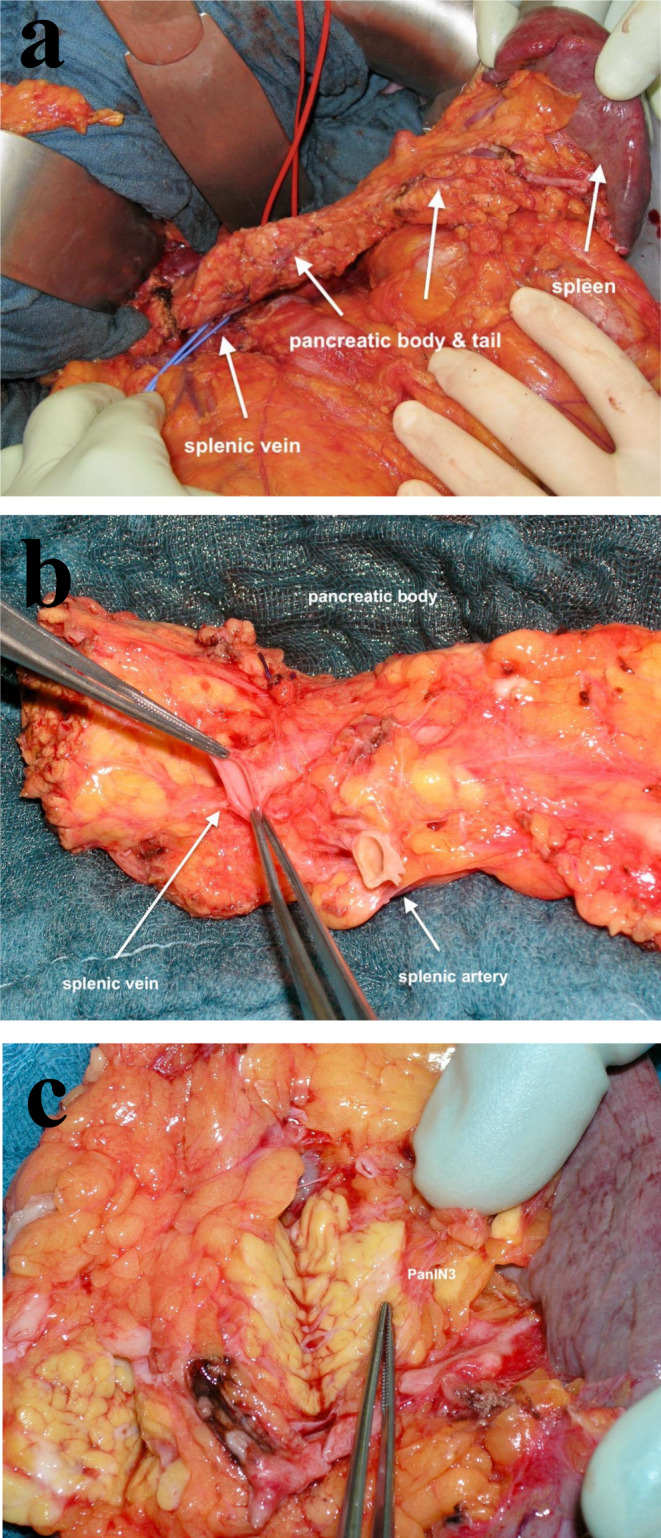
Total pancreatectomy for multiple IPMN, the patient was intended for an autologous islet transplantation and the pancreatic body was already prepared, final histopathology showed PanIN3 lesions and islet transplantation was not performed. (Department of Visceral-, Thoracic- and Vascular Surgery, Philipps-University Marburg). **A** Intraoperative situs of distal pancreatic resection as part of total pancreatectomy. **B** Preparation for islet transplantation. **C** Suspicious lesion, later classified as PanIn3

To avoid postoperative insulin substitution the isolation of about 160.000 islet cells from the resected pancreas and the autotransplantation of roughly 2000 islet cells/kg body weight via the portal vein is mandatory [27]. Prerequisite of a successful TPAIT is fast processing of the resected pancreas, including a detailled histopatholgical analysis. In 2014, the participants of PancreasFest [28] published recommendations for TPAIT. According to these criteria pancreatic malignancy and high-grade precursor lesions have been considered an absolute contraindication for islet autotransplantation, because of the risk to disseminate cancer cells through the infusion of islets, which may still contain some exocrine cells even after purification. In 2016, Balzano et al. postulated criteria of extended indications for TPAIT [29] based on the experience with 31 patients with malignant sporadic pancreatic or periampullary neoplasms. Ex novo liver metastases following TPAIT were noted in only 3 patients and relapse was observed in 5 (12.9%) of 31 patients after median 2.5 years after TPAIT. The comparison of overall survival and disease-/progression-free survival of patients with PDAC treated with islet autotransplantation with those of patients with PDAC who had surgery without islet autotransplantation in the same period of time, the TPAIT group had a better survival at a similar stage of disease. At present, however, the use of TPAIT in malignant or high grade premalignant remains controversial, and thus is currently not standard management [30]. The FaPaCa group has intended TPAIT in 2 IAR (Fig. 2). However, both IAR had multifiocal PanIN3-lesions in the processed resected pancreas, so that the interdisciplinary board as well as the ethic committee voted against autoinfusion of the prepared islet cells (unpublished data). Large, prospective multicentric trials are needed to assess the long-term oncological results in the setting of hereditary PDAC.

### Total pancreatectomy with pancreas transplantation

After total pancreatectomy in IAR with high grade PanIN2/3 lesions there is also the theroretical possibilty for pancreas transplantation. Charpentier et al. already reported in 2004 a 42-years old male IAR of a FPC family, who underwent total pancreatectomy for multifocal PanIN2 and 3 lesions. One year after pancreatectomy an allogenic pancreas transplantation was performed [31]. Fifteen months post transplantation the patient was free of insulin and showed no evidence of a PDAC or metastases on imaging. Pancreas transplantation in the setting of hereditary PDAC, however, remains only a theoretical option due to the lack of organs and all the uncertainties with precursor tumor cell spreading under the required immunosuppression.

### Complications after surgery for hereditary PDAC

Several cohort studies have shown that the morbidity rate of major pancreatic resections in IAR, are similar with up to 30% clinically relevant (Clavien-Dindo ≥ 3) complications to those of patients with resected sporadic PDAC or high-grade IPMN (Table 2) The mortality of resected IAR in reported series [8, 32–34] and in a recent meta-analysis [35], however, is 0%, and thus lower than in sporadic PDAC. This might be due to the strong selection of IAR for pancreatic surgery in interdisciplinary expert programs.

**Table 2:** Postoperative complications and mortality after pancreatic surgery in IAR as part of a surveillance program

Study	No. IAR	surgical resection	Type of surgery	Clinically relevant complications	mortality
Vasen et al. 2016 [33]	411	7.3%	TP *n* = 7 PPD *n* = 5 DP *n* = 18	13.3% (4/30)	0
Canto et al. 2020 [34]	354	13.6%	TP *n* = 6 PPD *n* = 16 DP *n* = 26	35.4% (17/48)	0
Bartsch et al. 2021 [18]	295	5.4%	TP *n* = 7 PPD *n* = 2 DP *n* = 7	31% (5/16)	0
Dbouk et al. 2022 [11]	1461	1.1%	PPD *n* = 4 DP *n* = 7	0	0
Paiella et al. 2023 [36]	524	2.1%	TP *n* = 6 PPD *n* = 2 DP n_2 palliative surgery *n* = 1	n.a.	0

TP - total pancreatectomy; PPD - partial pancreatoduodenectomy; DP - distal pancreatectomy

### Meeting the goal of surgery in hereditary PDAC

The goal of PDAC screening in IAR to detect PDAC at UICC stage I or its high-risk precursor neoplasms and consequently should early surgery of these screening detected lesions prolong survival. Vasen et al. [33] provided first evidence that surveillance of *CDNK2A* variant carriers is relatively successful, detecting most PDAC (75%) at a resectable stage with a 5-year survival rate of 24%. A retrospective examination of 16 international surveillance programs, however, showed that 35 of 41 screening detected PDAC were either unresectable (*n* = 14) or had advanced tumors with lymph node metastases (*n* = 21) [37]. In a recent Dutch study only 3 of 10 IAR with PDAC from FPC families met the postulated goal of screening, namely early PDAC confined to the pancreas without metastases, but the resectability rate was 60% with a median survival of 21 months for resected cases [33]. Currently, in the FaPaCa cohort, also 3 of 4 IAR with PDAC had cancers with lymph node metastases, but all 4 PDAC could be resected with median survival of 32.5 months. Thus, the goal of PDAC screening was not fulfilled in most FPC screening programs. The reported resectability and postoperative survival rates, however, compare favorably to that of sporadic PDAC outside surveillance programs, although longer survival partly be due to lead time bias cannot be excluded (Tables 3, 38 and 39).

**Table 3:** Histopathological outcomes of surgery and survival from recent available surveillance programs with over 200 IAR

Study	No. IAR	overall resection rate	advanced, metastasized PDCA	survival	resected PDAC	Survival	resected PanIn3	Survival	resected HG-IPMN	Survival
Bartsch et al. 2016 [8]	253	8.3% (21/253)	0	n.a.	2 1xUICC I 1xUICC IIb	17 months (NED) 38 months (AWD)	3	70 months NED) 61 months (NED) 16 months (NED)	1	42 months (NED)
Vasen et al. 2016 [33]	411	7.3% (30/411)	4	15 months (DOD) 10 months (DOD) 8 months (DOD) 6 months (DOD)	11 5x UICC I 4xUICC II 2x UICC III	5-y-survival 24% 25 months (A) 21 months (DOD) 73 months (A) 6 months (A) 17 months (DOD) 35 months (DOD) 18 months (DOD) 36 months (A) 38 months (DOD) 4,5 months (DOD) 22 months (DOD)	3	55 months (A) 49 months (A) 16 months (A)	1	30 months (A)
Canto et al. 2020 [34]	354	13.6% (48/354)	3	n.a.	11 2xUICC I 2xUICC IIa 6xUICC IIb 1xUICC IV	1-y-survival 90% 5-y-survival 60%	6	1-y-survival 100% 5-y-survival 100%	4	1-y-survival 100% 5-y-survival 100%
Dbouk et al. 2022 [11]	1461	1.1% (16/1461)	2	24 months (A) 17 months (DOD)	8 4xUICC Ia (1x neoadjuvant chemotherapy*) 3xUICC Ib (1x neoadjuvant chemotherapy*) 1xUICC IIb	26 months (A) 5 months (A) 23 months (A) 10 months (A) 31 months (A) 49 months (A) 46 months (DOD) 42 months (A)	1	34 months (A)	2	6 months (A) 37 months (A)
Klatte et al. 2022 [40]	347	10.4% (36/347)	9**	15 months (DOD 10 months (DOD) 8 months (DOD) 9 months (DOD) 27 months (DOD) 16 months (DOD) 15 months (DOD) 1 months (DOD) 12 months (A)**	22 5xUICC Ia (1x neoadvant chemotherapy) 2xUICC Ib 7xUICC IIa 6xUICC IIb (1x neoadvant chemotherapy) 2xUICC III (1x neoadvant chemotherapy)	overall 5-y-survival 44.1% 87 months (A) 57 months (A) 47 months (A) 12 months (A) 23 months (A) 21 months (DOD) 40 months (A) 153 months (A) 34 months (DOD) 31 months (DOD) 20 months (DOD) 63 months (A) 51 months (A) 36 months (A) 17 months (DOD) 115 months (A) 22 months (DOD) 21 months (DOD) 38 months (A) 31 months (A) 3 months (DOD) 21 months (DOD)	0	n.a.	0	n.a.
Paiella et al. 2023 [36]	524	2.1% (11/524)	3	2 months (DOD) 12 months (DOD) 2 months (DOD)	5 4xUICC I 1xUICC III	30 months (NED) 18 moths (DOD) 28 months (NED) 1 months (NED) 14 months (DOD)	1	26 months (NED)	0	n.a.

NED = no evidence of disease, DOD = dead of disease, DURC = dead of unrelated cause, A = alive, AWD = alive with disease; PDAC = pancreatic ductal adenocarcinoma; PanIN = pancreatic intraepithelia neoplasia; IPMN = intraductal papillary mucinous neoplasia; BD-IPMN = branch duct type intraductal papillary mucinous neoplasia

*in 2 cases UICC Ia and b after neoadjuvant therapy, pretherapeutic stage Ia and IIa

** one case cT1b cN0 cM0 treated with chemoembolization & microwave ablation

Currently applied indications, which are mainly based on EUS and MRI, carry a risk of surgical overtreatment. A previous meta-analysis reported that 67% (198/257) of IAR had pancreatic resections for non-target lesions [41]. In a previous Dutch study 30–40% of IAR [33] and in the FaPaCa cohort [17] at least 25% of IAR underwent unnecessary pancreatic resections. A very recent meta-analysis based on 5027 IAR in 23 studies stated a pooled prevalence of low-yield surgery of only 2.1% [95%CI 0.9–3.7]. The temporal analysis showed that the rate of low-yield surgeries decreased in the last decade and stabilized at around 1% [35]. This reported low rate of low-yield surgery in IAR cannot be confirmed by the authors experience.

### Psychological factors regarding surgery in IAR

The Dutch colleagues reported 2020 about the burden of intensified surveillance and surgery in IAR [29]. 298 IARs were under surveillance, 15 of them (5.0%) underwent surgery. 67% (10/15) were interviewed a median of 43 months after surgery. Most patients felt surgery justified (70%). All IAR would have voted for surgery again regardless of whether histopathological results showed PDAC (10%), precursors (50%) or benign findings (30%) and the postoperative recovery was uneventful or with complications [42].

## Conclusion

The goal of surgery in hereditary PDAC is the early resection of PDAC at UICC stage I or its high-risk precursors to prolong survival in IAR under surveillance. Indication for surgery for IAR with proven PDAC or suspicious solid lesion(s) and also for cystic lesions should be based on the established guidelines for individuals without a known familial/genetic risk and experts’ recommendations on FPC. Diameter and number of cysts should be considered and assessed more strictly in IAR, the same applies for potential prophylactic extent of resection when detecting precursors in the resected specimen or in the resection margins.

## Data Availability

No datasets were generated or analysed during the current study.
